# Severe Sepsis in Two Ugandan Hospitals: a Prospective Observational Study of Management and Outcomes in a Predominantly HIV-1 Infected Population

**DOI:** 10.1371/journal.pone.0007782

**Published:** 2009-11-11

**Authors:** Shevin T. Jacob, Christopher C. Moore, Patrick Banura, Relana Pinkerton, David Meya, Pius Opendi, Steven J. Reynolds, Nathan Kenya-Mugisha, Harriet Mayanja-Kizza, W. Michael Scheld, for the Promoting Resource-limited Interventions for Sepsis Management in Uganda (PRISM-U) Study Group

**Affiliations:** 1 Division of Allergy and Infectious Diseases, Department of Medicine, University of Washington, Seattle, Washington, United States of America; 2 Division of Infectious Diseases and International Health, Department of Medicine, University of Virginia Health System, Charlottesville, Virginia, United States of America; 3 Department of Community Health, Masaka Regional Referral Hospital, Masaka, Uganda; 4 Infectious Diseases Institute, Faculty of Medicine, Makerere University, Mulago Hospital Complex, Kampala, Uganda; 5 Rakai Health Sciences Program, Kalisizo, Uganda; 6 Department of Intramural Research, National Institutes of Health, Bethesda, Maryland, United States of America; 7 Johns Hopkins University School of Medicine, Baltimore, Maryland, United States of America; 8 Uganda Ministry of Health, Kampala, Uganda; 9 Faculty of Medicine, Makerere University, Mulago Hospital Complex, Kampala, Uganda; University of Giessen Lung Center, Germany

## Abstract

**Background:**

Sepsis likely contributes to the high burden of infectious disease morbidity and mortality in low income countries. Data regarding sepsis management in sub-Saharan Africa are limited. We conducted a prospective observational study reporting the management and outcomes of severely septic patients in two Ugandan hospitals. We describe their epidemiology, management, and clinical correlates for mortality.

**Methodology/Results:**

Three-hundred eighty-two patients fulfilled enrollment criteria for a severe sepsis syndrome. Vital signs, management and laboratory results were recorded. Outcomes measured included in-hospital and post-discharge mortality.

Most patients were HIV-infected (320/377, 84.9%) with a median CD4+ T cell (CD4) count of 52 cells/mm^3^ (IQR, 16–131 cells/mm^3^). Overall mortality was 43.0%, with 23.7% in-hospital mortality (90/380) and 22.3% post-discharge mortality (55/247). Significant predictors of in-hospital mortality included admission Glasgow Coma Scale and Karnofsky Performance Scale (KPS), tachypnea, leukocytosis and thrombocytopenia. Discharge KPS and early fluid resuscitation were significant predictors of post-discharge mortality. Among HIV-infected patients, CD4 count was a significant predictor of post-discharge mortality.

Median volume of fluid resuscitation within the first 6 hours of presentation was 500 mLs (IQR 250–1000 mls). Fifty-two different empiric antibacterial regimens were used during the study. Bacteremic patients were more likely to die in hospital than non-bacteremic patients (OR 1.83, 95% CI = 1.01–3.33). Patients with *Mycobacterium tuberculosis* (MTB) bacteremia (25/249) had higher in-hospital mortality (OR 1.97, 95% CI = 1.19–327) and lower median CD4 counts (*p* = 0.001) than patients without MTB bacteremia.

**Conclusion:**

Patients presenting with sepsis syndromes to two Ugandan hospitals had late stage HIV infection and high mortality. Bacteremia, especially from MTB, was associated with increased in-hospital mortality. Most clinical predictors of in-hospital mortality were easily measurable and can be used for triaging patients in resource-constrained settings. Procurement of low cost and high impact treatments like intravenous fluids and empiric antibiotics may help decrease sepsis-associated mortality in resource-constrained settings.

## Introduction

Sepsis in high income countries (HICs) accounts for a significant burden of disease and mortality and has been implicated as the leading cause of non-cardiac death amongst critically ill patients in these settings [1]–[4]. Similar epidemiologic data on sepsis in low and middle income countries (LMICs) are unavailable. Several studies from sub-Saharan Africa (SSA) reveal an association between HIV infection and an increased likelihood of bacteremia and mortality [5]–[12]. While the ultimate cause of death is not identified in these patients, it is likely that a large proportion succumb to severe sepsis [4].

In HICs, algorithmic approaches to sepsis management which focus on early diagnosis and antimicrobial treatment, aggressive fluid resuscitation and concomitant monitoring of such parameters as central venous pressure, central venous oxygen saturation, and hematocrit can decrease sepsis-associated mortality [13], [14]. In LMICs, data regarding the management and outcomes of sepsis syndromes are limited [15]. Factors thought to contribute to poor outcomes of critically ill patients in these settings include limitations due to cost, deficiency of diagnostic laboratories, microbiologic and radiologic capabilities and delayed presentation of severely sick patients [6], [16], [17]. Notably, a retrospective hospital chart review from Zambia revealed that 79 (86%) of 92 hypotensive patients with suspected sepsis received no intravenous (IV) fluid resuscitation [18]. Additionally, a study of bacteremic children in Tanzania highlighted increased mortality when empiric antibiotics were discordant with antibiotic susceptibility profiles [19].

The scarcity of data on this topic in developing countries necessitates further studies to evaluate cost-effective approaches to sepsis in these settings [20]. While HIC approaches to sepsis are not feasible in the current resource-constrained environment of SSA, the timely provision of IV fluid resuscitation and empiric antibiotics alone may improve outcomes [21]. We conducted the first prospective observational study in an LMIC of severely septic adults admitted to the medical wards of two government hospitals in Uganda. The objectives of the study were to 1) identify clinical predictors for in-patient and post-discharge mortality among hospitalized Ugandan patients with severe sepsis, and 2) describe the epidemiology, management, and etiology of sepsis in this setting. Given the resource constraints in these hospitals, we paid particular attention to potentially cost-effective interventions such as initial fluid resuscitation and empiric antibiotic administration.

## Methods

### Ethics statement

Approval was obtained from the University of Virginia Institutional Review Board, Mulago Hospital Office of Director, Makerere University Faculty of Medicine Research Ethics Committee and Infectious Disease Institute Scientific Research Committee, and Uganda National Council of Science and Technology.

### Site descriptions

Mulago Hospital is a 1500-bed national referral hospital in Kampala, Uganda. Approximately 64% of medical ward patients are infected with human immunodeficiency virus type 1 (HIV) [22]. Masaka Regional Referral Hospital (MRRH) is a 330-bed regional referral hospital in Masaka, Uganda (125 kilometers southwest of Kampala) serving five surrounding districts. Both Mulago and Masaka Hospitals exist under the auspices of the Ministry of Health and were selected out of convenience as representations of a national and regional referral government hospital in Uganda.

### Site resource capacity

In HICs, sepsis management requires close monitoring of central venous pressure, mean arterial pressure (MAP), urine output, central venous oxygen saturation, peripheral oxygen saturation, and hemoglobin [14]. During the course of the study, the only parameters measurable were MAP and hemoglobin. Pulse oximeters were sparse at Mulago and not available at MRRH. At Mulago Hospital, one oxygen tank existed for the emergency medicine ward (∼50 beds) from which nasal cannulae were used to simultaneously provide oxygen for up to 5 hypoxic or tachypneic patients. No oxygen tanks were available on the medical wards in MRRH. Since vasopressor or inotropic medications were scarce, cardiovascular support medications, when available (e.g., dopamine and epinephrine), were rarely utilized, especially on the medical ward. There was a 5-bed intensive care unit (ICU) present at Mulago Hospital but not one at MRRH. The Mulago ICU was mainly available for surgery and private patients. Thus, adult medical patients, especially those with HIV and tuberculosis, were rarely admitted to the ICU.

### Patient recruitment

From July 2006 until November 2006, patients were enrolled consecutively on weekdays during daytime hours from the Accident and Emergency Departments of both hospitals. Patients were included if they were ≥18 years of age, admitted to a medical ward and had: 1) a suspected infection, 2) ≥2 of the following: axillary temperature (TMP) >37.5°C or <35.5°C; heart rate (HR) >90 beats/min; or respiratory rate (RR) >20 breaths/min and 3) systolic blood pressure (SBP) ≤100 mmHg. Patients were excluded if they had an acute cerebrovascular event, gastrointestinal hemorrhage, or required admission to a non-medical ward. Written informed consent was obtained from each patient; if patients were unable to provide consent, written consent was obtained from their attendant. Attendants were most often comprised of the patient's next of kin but also included friends or neighbors. Patients who were unable to provide consent and not accompanied by an attendant were not enrolled in the study.

### Data collection

Patients were enrolled based on parameters measurable at the time of evaluation. These parameters included HR, TMP, RR, and blood pressure recorded at enrollment and 6 and 24 hours afterwards. White blood cell (WBC) concentration was not included in the entry criteria due to unavailability at the time of admission. WBC concentration, however, was measured and used to ultimately assess whether patients fulfilled previously established criteria for sepsis [23]. Along with Glasgow Coma Scale (GCS), admission Karnofsky Performance Status (KPS) was used to assess admission morbidity; discharge KPS was used to assess morbidity at discharge [24]. Use of KPS as a morbidity assessment scale has been well described in oncology literature and has been shown to predict survival among Ugandan HIV-infected patients within the outpatient setting [25], [26].

While the in-hospital medical team was responsible for all clinical decisions regarding patient management, the study team recorded patient management details until discharge or death. Specifically, the study team recorded the amount of IV fluid resuscitation administered in the first 6 and 24 hours and type of empiric antimicrobial agents given at presentation. In addition, information regarding blood transfusions and radiologic studies was recorded when available. Appropriate empiric antibiotic therapy was defined as use of a regimen to which the isolate was found to be sensitive in susceptibility testing. All treatments given were paid for by either the hospital or, in cases (e.g., blood transfusions) where the hospital could not provide the treatment, by the patient or family members.

The primary outcome measure was mortality, analyzed as in-hospital and post-discharge mortality (among patients who survived to discharge). Attempts were made to contact patients 30 days after discharge by telephone for information regarding outpatient survival. If patients did not have access to a telephone, they were offered reimbursement for the cost of transportation to return to the Accident and Emergency Department for a post-discharge, 30-day assessment.

### Laboratory evaluation, reliability and validity

Due to the lack of a centralized laboratory for study patients, the services of several organizations were utilized for laboratory testing. HIV antibody testing was performed on site at each hospital using Determine [Abbott Laboratories, Tokyo, Japan] and Statpak [Chembio Diagnostic Systems, Inc, Medford, USA]. If results of the initial antibody tests were equivocal, Unigold [Trinity Biotech plc, Bray, Ireland] was used for a tie breaker. Malaria thick smears were also performed, as previously described [27]. Point-of-care portable whole blood lactate (PWBL) (Accutrend portable lactate analyzer; Sports Resource Group, Inc., USA) testing was performed on site using blood collected by fingerstick, as previously described [28].

### Mulago site

At the Mulago site, Ebenezer Limited Clinical Laboratory (Kampala, Uganda), accredited by the South African National Accreditation System (SANAS), provided results for general laboratory evaluations including complete blood counts (CBC) with differentials (Sysmex KX-21, TOA Medical Electronics, Kobe, Japan); CD4+ T cell (CD4) counts (FACSCount System, BD Biosciences, San Jose, CA); serum lactic acid and bicarbonate (Diagnostic Systems International, Holzheim, Germany); cortisol (BioMerieux s.a., Marcy L'Etoile, France); albumin, blood urea nitrogen and creatinine (Cobas Mira Plus CC Analyzer, Roche Diagnostics, North America); and sodium, potassium, and chloride (HumaLyte, Human Diagnostics Worldwide, Wiesbaden, Germany).

Aerobic cultures were performed at the Makerere University microbiology laboratory; mycobacterial cultures were performed at the Joint Clinical Research Center (Kampala, Uganda). Both these laboratories have enrolled in International Organization for Standardization (ISO) processes and participate in World Health Organization (WHO) External Quality Assurance for microscopy, culture and drug susceptibility testing. Standard American Type Culture Collection (ATCC) isolates were used for quality control.

### Masaka site

At MRRH, fewer facilities exist for laboratory testing than at Mulago Hospital. As a result, only CBCs (Humacount, Human Diagnostics Worldwide, Wiesbaden, Germany) and CD4 testing (Guava Technologies, Hayward, CA) were performed at MRRH using nearby organizations with laboratory capabilities.

Aerobic cultures were obtained at the nearby Rakai Health Sciences Project (RHSP) which participates in external quality assurance programs provided by the College of American Pathologists (CAP) and maintained satisfactory performance throughout the study period on all culture and antibiogram surveys. RHSP provides quality assurance and control for laboratories in the area including the Uganda Cares Initiative, Masaka where CD4 counts were performed and AID Child, Masaka, where CBCs were performed.

### Microbiology

Once enrolled at Mulago Hospital, 5–7 mLs of blood were aseptically inoculated into 2 aerobic blood culture bottles (Liofilchem s.r.l. Bacteriology products, Roseto, Italy). Subculturing and species identification were performed using standard methods. Sensitivities were determined based on turbidity compared with a MacFarland Standard and inoculated on Mueller-Hinton agar (Liofilchem s.r.l. Bacteriology products, Roseto, Italy) for evaluation of zone diameters associated with antimicrobial discs.

For Mulago patients only, another 3–5 mLs of blood were aseptically inoculated into BACTEC Myco/F Lytic culture vials (Becton Dickinson, Sparks, Maryland, USA) for mycobacterial cultures. Mycobacteria detected in blood were incubated and monitored in a BACTEC 9120 instrument (Becton Dickinson, Sparks, Maryland, USA). Positive mycobacterial cultures were further identified as *Mycobacterium tuberculosis* (MTB) complex by IS6110 PCR amplification using a PTC-200 Peltier Thermal Cycler (MJ Research, Reno, NV, USA).

At MRRH, ten mLs of blood were aseptically inoculated into two aerobic blood culture bottles (BBL Septi-Chek, Becton Dickinson, Sparks, MD). BBL Septi-Chek Slides (Becton Dickinson, Sparks, MD) were then attached to culture bottles and observed for primary growth which was subcultured and speciated using standard methods. Susceptibility testing was performed using the ATB system (Biomerieux, France). To determine susceptibility to chloramphenicol, Mueller-Hinton agar disc diffusion was used on Gram negative isolates as this antibiotic is not included in the ATB Gram negative panel.

Bacteremia was defined as bacterial isolation from one or more blood culture bottles. Cultures growing coagulase-negative staphylococci were not included in the analyses.

### Statistical analysis

Data were entered into a spreadsheet using Epi-Info (version 6.04d, Centers for Disease Control and Prevention, USA) and analyzed using SPSS software (version 15.0, SPSS Inc., USA). Descriptive statistics were reported as mean and standard deviation for normally distributed variables and median and interquartile range (IQR) for variables not normally distributed. The χ2 analysis of variance was used to compare differences for categorical variables. Odds ratios (OR) with 95% confidence intervals (CI) were calculated. The Student's paired sample t-test was used to compare mean differences between continuous variables. The Mann-Whitney U test was used to compare non-parametric continuous variables (e.g., CD4 count). Statistical significance was defined as *p*<0.05. Variables with clinically relevant cut-off points were dichotomized.

In order to determine independent predictors of in-hospital mortality for the general study population, numerous variables were assessed including demographic characteristics (sex, mean age, marital status, education and hospital site), measurements of clinical status (GCS<15, admission KPS, fulfillment of ≥3 SIRS criteria, HR>110 bpm, RR>30 brpm, TMP>37.5 C, TMP<35.5 C, MAP<50 mmHg); laboratory studies (aerobic culture positivity, HIV-1 status, WBC>12,000 cells/mL, WBC<4,000 cells/mL, malaria blood smear positivity, platelets<150,000 cells/mL, and level of hemoglobin); and management (volume of fluid provided at 6 and 24 hours (≥1 L vs.<1 L), time to administration of fluid (<1 hour vs. ≥1 hour), whether or not empiric antibiotics were administered, and time to administration of empiric antibiotics (<1 hour vs. ≥1 hour). Additional covariates were assessed to determine independent predictors of post discharge mortality including discharge KPS and length of hospitalization.

Univariate analyses were used to identify which variables with a *p*-value less than 0.20 would be included in the multivariate models. Stepwise backward logistic regression was used to determine whether these covariates were independent predictors of mortality; covariates were eliminated when *p*-values were greater than or equal to 0.05.

The same methods were used to assess predictors of in-hospital and post-discharge mortality among HIV-infected patients from both hospital sites. Covariates assessed at the univariate level included CD4 count along with the same variables evaluated in the generalized patient models.

For patients from the Mulago Hospital site with additional laboratory testing, univariate analyses were used to assess whether patients' laboratory results at admission were related to in-hospital and post discharge 30-day mortality. Covariates significant at the *p*<0.20 level were entered into a multivariate model which adjusted for covariates found to be significant independent predictors of in-hospital and post-discharge mortality at Mulago Hospital.

## Results

### Background Characteristics

**Figure 1:**
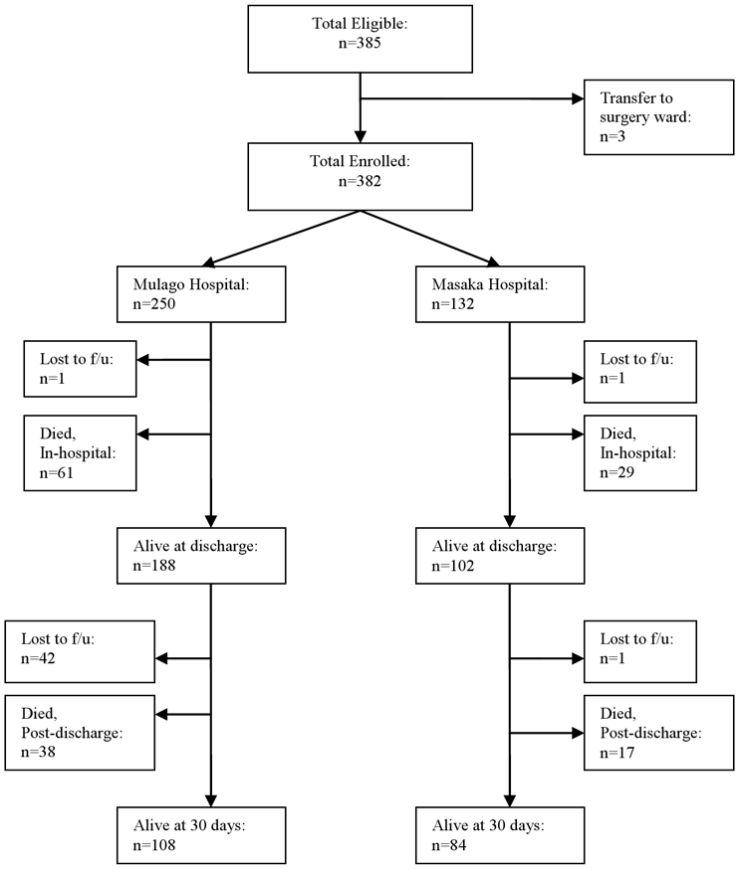
Flow diagram for study eligibility and follow-up to 30 days post-discharge.

Of 385 patients eligible for enrollment, 382 patients were enrolled (250 from Mulago Hospital, 132 from MRRH). Three patients were excluded because of transfer to a surgery ward (see Figure 1). Two patients absconded during their hospitalization and were lost to follow up.

The majority of patients enrolled were ARV-naïve, HIV-infected women (see Table 1). The most frequent chief complaints at the time of admission were fever (39.2%, 149/380), cough (19.6%, 75/380), and diarrhea (11.0%, 42/380). Attendants accompanied 91.1% (348/382) of patients. While the majority of attendants were comprised of next of kin, 41 of 348 patients were accompanied by friends or neighbors.

**Table 1:** Descriptive characteristics of hospital sites.

Characteristic	Combined (n = 382)*	Mulago (n = 250)*	Masaka (n = 132)*
***Demographics:***			
Age in years; mean (SD)	34.8 (11.2)	34.3 (10.51)	35.6 (12.3)
Number female (%)	226 (59.2%)	149 (59.6%)	77 (58.3%)
***Inclusion criteria:***			
Number fulfilling ≥3 SIRS criteria (%)	317 (83.2%)	212 (84.8%)	105 (80.2%)
Admit temp in °C; mean (SD)	37.6 (1.5)	37.7 (1.6)	37.4 (1.4)
Admit HR in beats/min; mean (SD)	120.1 (20.7)	122.3 (20.7)	115.6 (19.8)
Admit RR in breaths/min; mean (SD)	36.9 (11.8)	37.4 (12.1)	35.9 (11.3)
Admit WBC in cells/mL; mean (SD)	7478.5 (6912.5)	6926.9 (5962.6)	8554.5 (8525.5)
Admit MAP in mmHg; mean (SD)	59.5 (16.7)	56.8 (17.9)	64.6 (12.7)
Admit SBP in mmHg; mean (SD)	81.9 (15.8)	79.2 (16.8)	87.1 (12.1)
Admit DBP in mmHg; mean (SD)	48.15 (18.5)	45.37 (19.9)	53.40 (14.3)
***HIV descriptors:***			
Number HIV-infected (%)	320 (84.9%)	213 (85.9%)	107 (82.9%)
CD4 count in lymphocytes/mm^3^; median (IQR)†	52.0 (16.0–131.0)	42.0 (10.2–103.5)	65 (25.0–98.0)
Number aware of HIV status (%)†	216 (67.5%)	154 (72.3%)	62 (57.9%)
Number on HAART (%)†	38 (11.9%)	28 (13.1%)	10 (9.3%)
Number within WHO, stage IV (%)†	179 (57.4%)	128 (62.4%)	51 (47.7%)
***Other Clinical Variables:***			
Admit Karnofsky Performance Score; mean (SD)	46.4 (16.6)	43.2 (16.1)	52.3 (16.0)
Portable whole blood lactate in mmol/L; mean (SD)∞	3.8 (2.5)	4.1 (2.6)	3.7 (2.6)
Number with positive malaria blood smear (%)	51 (13.5%)	24 (9.6%)	27 (20.9%)

* Due to the occurrence of missing data found in <2% of the variables, numbers may not add up to total n.

† Denominator is Number HIV-infected.

∞ Portable whole blood lactate levels were not obtained on all patients [combined (n = 194); Mulago (n = 70); Masaka (n = 124)].

### Mortality

From hospital census reports during the study period, all-cause in-hospital mortality for the adult medical wards was 15.4% (1054 of 6856 patients) [29], [30]. Background post-discharge mortality data were not available from either hospital site. Of the 380 patients followed in this study, ninety (23.7%) died while hospitalized. Median length of hospital stay for the remaining 290 patients who survived to discharge was 6 days (IQR, 3–10). Of these patients, 43 (14.8%) were lost to follow-up after discharge; the majority (98%) were from Mulago Hospital.

Fifty-five (22.3%) of the remaining 247 patients died within 30 days of discharge. Thus, 145 (43.0%) of 337 patients contributed to overall mortality with the majority of deaths occurring soon after hospital admission. Of the 145 total deaths, 15 (10.3%) occurred in the first day, 42 (30%) occurred within 3 days after admission, 70 (48.3%) occurred within 7 days after admission, and 105 (72.4%) occurred within 28 days after admission.

### Clinical Correlates of Mortality

**Figure 2:**
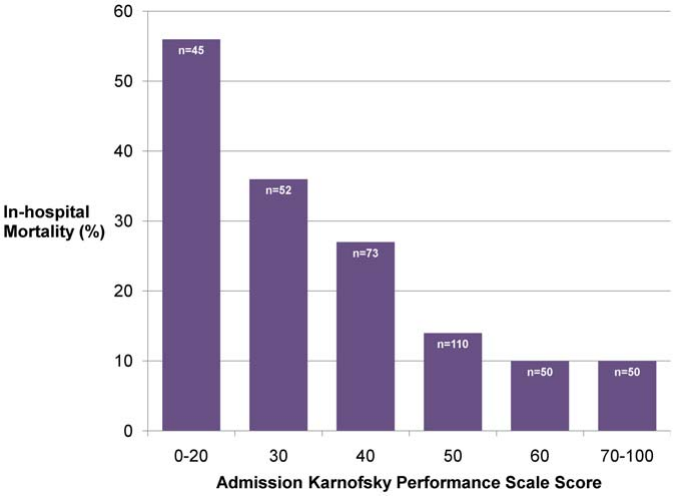
In-hospital mortality and admission Karnofsky Performance Scale score. [*Numbers in bars represent number of patients.]

Univariate analysis was used to assess the predictive value of variables with respect to in-hospital mortality in the general study patient population (see Table 2). In multivariate analysis, independent, significant predictors of in-hospital mortality (see Table 3) included GCS<15, decreased admission KPS (see Figure 2), RR>30 breaths/min, WBC>12,000 cells/mL, and platelets<150,000 cells/mL. These variables together explained 22% (Nagelkerke R^2^) of the variance associated with in-hospital mortality.

**Table 2:** Univariate analysis of clinical predictors for in-hospital mortality.

Variable	Subcategory	Died (n = 90)*	Survived (n = 290)*	OR (95% CI)	*p*
***Patient Demographic Characteristics***					
Female [n (%)] *Female*		51 (56.7)	173 (59.7)	0.9 (0.5–1.4)	0.62
Age [mean years (SD)]		35.3 (10.4)	34.6 (11.3)	n/a	0.64
Marital Status [n (%)]	*Married or in Relationship*	36 (40.9)	151 (52.8)	0.6 (0.4–1.0)	0.05
	*Unmarried or not in Relationship*	52 (59.1)	135 (47.2)		
Education [n (%)]	*None*	10 (4.4)	22 (7.7)	0.9 (0.6–1.3)	0.73
	*Primary School*	53 (60.2)	180 (63.2)		
	*Secondary School*	24 (27.3)	78 (27.4)		
	*University*	1 (1.1)	5 (1.8)		
Hospital Site [n (%)]	*Mulago*	61 (67.8)	188 (64.8)	1.1 (0.7–1.9)	0.61
	*Masaka*	29 (32.2)	102 (35.2)		
***Medical Status at Admission***					
Glasgow Coma Score <15 [n (%)]		17 (18.9)	15 (5.2)	4.3 (2.0–9.0)	<0.001
Karnofsky Score [mean (SD)]		37 (17)	49 (16)	n/a	<0.001
≥3 SIRS Criteria [n (%)]		75 (83.3)	241 (83.1)	1.0 (0.5–1.9)	0.96
HR>110 beats/min [n (%)]		70 (78.7)	190 (65.7)	1.9 (1.1–3.4)	0.02
RR>30 breaths/min [n (%)]		73 (82)	179 (62.2)	2.8 (1.5–5.0)	0.001
Temp>37.5°C [n (%)]†		52 (58.4)	168 (58.7)	1.3 (0.8–2.3)	0.33
Temp<35.5°C [n (%)]†		16 (18)	28 (9.8)	2.4 (1.1–5.3)	0.02
MAP<50 mmHg [n (%)]		24 (27.9)	43 (14.9)	2.2 (1.2–3.9)	0.01
***Laboratory results***					
Positive Aerobic Cultures [n (%)]		14 (15.6)	33 (11.4)	1.4 (0.7–2.8)	0.29
HIV Positive [n (%)]		79 (89.8)	240 (83.3)	1.8 (0.8–3.8)	0.14
WBC>12,000 cells/mL [n (%)]∞		16 (19)	41 (14.5)	1.7 (0.9–3.4)	0.12
WBC<4,000 cells/mL [n (%)]∞		32 (38.1)	84 (29.7)	1.7 (1.0–2.9)	0.06
Hb [mean g/dL (SD)]		8.4 (4.0)	9.2 (3.1)	n/a	0.04
Platelets<150,000 cells/mL [n (%)]		35 (42.2)	70 (25.2)	2.2 (1.3–3.6)	0.003
Positive Malaria Smear [n (%)]		13 (14.6)	38 (13.1)	1.1 (0.6–2.2)	0.72
***Management***					
≥1 Liter IV Fluid Provided at 6 Hours [n (%)]		35 (38.9)	95 (32.8)	1.3 (0.8–2.1)	0.28
≥1 Liter IV Fluid Provided at 24 Hours [n (%)]		52 (62.7)	175 (62.7)	1.0 (0.6–1.6)	0.99
<1 Hour to Administration of Fluid [n (%)]		57 (64.0)	142 (49.0)	1.8 (1.1–3.0)	0.01
Empiric Antibiotics Administered [n (%)]		77 (86.5)	241 (84.3)	1.2 (0.6–2.4)	0.60
<1 Hour to Administration of Antibiotics [n (%)]		28 (31.5)	93 (32.6)	0.9 (0.6–1.6)	0.84

* Due to the occurrence of missing data found in <2% of the variables, numbers may not add up to total n.

† Compared to the normal range of temperature (i.e., 35.5–37.5°C).

∞ Compared to the normal range of WBC (i.e., 4,000–12,000 cells/ml).

n/a = not applicable.

**Table 3:** Multivariate analysis of clinical predictors for in-hospital mortality.

Variable	OR (95% CI)	*p*
Glasgow Coma Score <15	2.7 (1.2–6.3)	0.022
Mean Admission Karnofsky Score*	0.6 (0.5–0.8)	<0.001
RR>30 breaths/min	2.3 (1.2–4.3)	0.012
WBC>12,000 cells/mL∞	2.8 (1.3–6.0)	0.01
Platelets<150,000 cells/ml	2.0 (1.1–3.6)	0.022

* Per 10-unit increase.

∞ Compared to the normal range of WBC (i.e., 4,000–12,000 cells/ml).

Univariate analysis was also used to assess the predictive value of variables with respect to post-discharge mortality (see Table 4). A separate multivariate analysis showed that independent, significant predictors of post-discharge mortality were decreased discharge KPS and earlier fluid resuscitation (<1 hour vs. ≥1 hour) (see Table 5). These variables together explained 24% (Nagelkerke R^2^) of the variance associated with post-discharge mortality.

**Table 4:** Univariate analysis of clinical predictors for post-discharge mortality.

Variable	Subcategory	Died (n = 55)*	Survived (n = 192)*	OR (95% CI)	*p*
***Patient Demographic Characteristics***					
Female [n (%)]		38 (69.1)	109 (56.8)	1.7 (0.9–3.2)	0.10
Age [mean years (SD)]		32.8 (9.2)	35.6 (11.8)	n/a	0.12
Marital Status [n (%)]	*Married or in Relationship*	27 (50.0)	101 (53.4)	0.9 (0.5–1.6)	0.66
	*Unmarried or not in Relationship*	27 (50.0)	88 (46.6)		
Education [n (%)]	*None*	5 (9.3)	15 (8.0)	0.6 (0.4–1.0)	0.23
	*Primary School*	39 (72.2)	113 (60.1)		
	*Secondary School*	10 (18.5)	55 (29.3)		
	*University*	0 (0)	5 (2.7)		
Hospital Site [n (%)]	*Mulago*	38 (69.1)	108 (56.3)	1.7 (0.9–3.3)	0.09
	*Masaka*	17 (30.9)	84 (43.8)		
***Medical Status***					
Glasgow Coma Score <15 [n (%)]		1 (1.8)	13 (6.8)	0.2 (0.03–2.0)	0.16
Admit Karnofsky Score [mean (SD)]		41 (12)	52 (15)	n/a	<0.001
Discharge Karnofsky Score [mean (SD)]		59 (16)	73 (14)	n/a	<0.001
≥3 SIRS Criteria [n (%)]		45 (81.8)	162 (84.4)	0.8 (0.4–1.8)	0.65
Admit HR >110 beats/min [n (%)]		37 (67.3)	120 (62.8)	1.2 (0.6–2.3)	0.54
Admit RR >30 breaths/min [n (%)]		35 (64.8)	117 (61.3)	1.2 (0.6–2.2)	0.63
Admit Temp >37.5°C [n (%)]†		38 (69.1)	106 (55.8)	1.8 (0.9–3.6)	0.10
Admit Temp <35.5°C [n (%)]†		4 (7.5)	19 (10.0)	1.0 (0.3–3.6)	0.94
Admit MAP <50 mmHg [n (%)]		12 (21.8)	21 (11.1)	2.2 (1.0–4.9)	0.04
***Laboratory results***					
Positive Aerobic Cultures [n (%)]		6 (10.9)	27 (14.1)	0.7 (0.3–1.9)	0.54
HIV Positive [n (%)]		52 (94.5)	155 (81.6)	3.9 (1.2–13.3)	0.02
WBC>12,000 cells/mL [n (%)]∞		3 (5.6)	28 (15.1)	0.4 (0.1–1.3)	0.11
WBC<4,000 cells/mL [n (%)]∞		21 (38.9)	57 (30.6)	1.2 (0.6–2.4)	0.51
Hemoglobin [mean g/dL (SD)]		7.8 (0.3)	9.6 (0.2)	n/a	<0.001
Platelets<150,000 cells/mL [n (%)]		17 (31.5)	44 (24.3)	1.4 (0.7–2.8)	0.29
Positive Malaria Blood Smear [n (%)]		7 (12.7)	29 (15.2)	0.8 (0.3–2.0)	0.65
***Management***					
≥1 Liter IV Fluid Provided at 6 Hours [n (%)]		20 (36.4)	60 (31.3)	1.2 (0.7–2.4)	0.48
≥1 Liter IV Fluid Provided at 24 Hours [n (%)]		35 (66.0)	118 (62.8)	1.2 (0.6–2.2)	0.66
<1 Hour to Administration of Fluid [n (%)]		32 (58.2)	86 (44.8)	1.7 (0.9–3.1)	0.08
Empiric Antibiotics Administered [n (%)]		42 (76.4)	163 (85.8)	0.5 (0.2–1.1)	0.10
<1 Hour to Administration of Antibiotics [n (%)]		18 (32.7)	57 (30.2)	1.1 (0.6–2.1)	0.72
Duration of hospitalization [mean days (SD)]		8.8 (6.9)	7.5 (5.6)	n/a	0.15

* Due to the occurrence of missing data found in <2% of the variables, numbers may not add up to total n.

† Compared to the normal range of temperature (i.e., 35.5–37.5°C).

∞ Compared to the normal range of WBC (i.e., 4,000–12,000 cells/ml).

n/a = not applicable.

**Table 5:** Multivariate analysis of clinical predictors for post-discharge mortality.

Variable	OR (95% CI)	*p*
Discharge Karnofsky Score*	0.5 (0.4–0.7)	<0.001
Time to Administration of Fluid, <1 hour	2.7 (1.2–5.9)	0.015

* Per 10-unit increase.

A separate model including only HIV-infected patients (n = 320) showed that CD4 count (*p* = 0.012), discharge KPS (*p*<0.001) and early fluid resuscitation (*p* = 0.031) were significantly associated with post-discharge mortality (data not shown). CD4 count did not contribute to prediction of in-hospital mortality among HIV-infected patients.

### Fluid Resuscitation

Overall, only 46 (12%) of 381 patients received more than 1500 mLs of IV fluid within the first 6 hours of presentation. The median volume of IV crystalloid received within the first 6 hours was 500 mLs (IQR = 250–1000) and at 24 hours was 1000 mLs (IQR = 500–1500). Only 4 of 382 patients received a blood transfusion within their first 6 hours of presentation.

Given the minimal IV fluid provided overall, no survival benefit was observed across strata of fluid volume in the general study population. Of the 25 patients who had confirmed Gram negative bacteremia, however, overall mortality was significantly decreased in patients receiving at least 1 L of fluid when compared to patients receiving <1 L of fluid [20% (2/10) vs. 66.7% (10/15), *p* = 0.022; OR 0.1, 95% CI = 0.02–0.8].

Timing of fluid resuscitation was an important predictor of in-hospital mortality at the univariate level and post-discharge mortality at the multivariate level. About half (53%) of patients received at least some IV fluid within the first hour of presentation while 28% waited between 1 to 6 hours; 14% of patients received no fluid within the first 6 hours of presentation. Patients receiving initial fluids in <1 hour from presentation were significantly more likely to die in-hospital than patients receiving fluids at or after 1 hour [28.6% (57/199) vs. 18.2% (33/181), *p* = 0.01; OR 1.8, 95% CI = 1.08–3.02]. When adjusted for significant predictors of in-hospital mortality, however, the relationship between time to fluid resuscitation and in-hospital mortality did not remain significant. To determine whether sicker patients were more likely to receive earlier fluid resuscitation, time to fluid resuscitation was evaluated in a multivariate analysis with significant predictors of in-hospital mortality comprising the model. Independent, significant predictors of early fluid resuscitation were admission KPS (OR 0.7 per 10-unit increase, 95% CI = 0.6–0.8) and platelets <150,000 cells/mL (OR 1.8, 95% CI = 1.1–2.9).

### Empiric Antibacterial Administration

**Figure 3:**
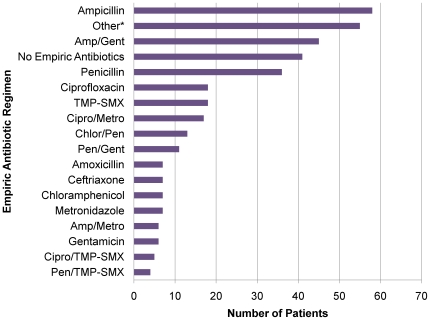
Frequency of separate empiric antibiotic regimens used. [*Represents 35 separate regimens used <1% of the time; Pen/Gent = Penicillin/Gentamicin; Pen/TMP-SMX = Penicillin/Trimethoprim-Sulfamethoxazole; Amp/Metro = Ampicillin/Metronidazole, Amp/Gent = Ampicllin/Gentamicin, Chlor/Pen = Chloramphenicol/Penicillin; Cipro/TMP-SMX = Ciprofloxacin/Trimethoprim-Sulfamethoxazole, Cipro/Metro = Ciprofloxacin/Metronidazole; TMP-SMX = Trimethoprim-Sulfamethoxazole]

During the course of the study, availability of antibacterial therapy at any given time was inconsistent. Accordingly, fifty-two different empiric antibacterial combinations were used by the admitting doctors caring for the study patients (see Figure 3). In total, 84.8% (319/376) of patients received some form of empiric antibacterials. There was no overall mortality benefit found between patients receiving any of the 52 available empiric antibacterial regimens compared to patients receiving no empiric antibacterials (23.8% vs. 22.6%, *p* = 0.85).

While 61% of these patients received antibacterials within the first 6 hours, only 37.8% received them within the first hour of presentation. Earlier administration of antibacterials (<1 hour vs. ≥1 hour) was neither predictive of in-hospital nor post-discharge mortality.

With respect to antibiotic susceptibilities, approximately 95% of *Salmonella* isolates were resistant to chloramphenicol and trimethoprim-sulfamethoxazole (TMP-SMX); none of the *Staphylococcus aureus* samples were resistant to oxacillin. In a subset of patients with positive aerobic cultures where susceptibilities were tested (n = 30), there was a trend towards decreased overall mortality in patients receiving appropriate versus inappropriate empiric antibacterials (18.2% vs. 50%, *p* = 0.074).

### Microbiology

Bacteremia was identified by aerobic and mycobacterial cultures in 72 (18.9%) of 381 patients. Patients with bacteremia were more likely to die while hospitalized than patients without bacteremia [33.3% (24/72) vs. 21.4% (66/308), *p* = 0.032; OR 1.83, 95%CI = 1.01–3.33]. Organisms grown from aerobic cultures were both Gram negative and Gram positive (n = 48) with the most frequently isolated organisms being non-typhoidal *Salmonella* (20%), *Staphylococcus aureus* (12%) and *Streptococcus pneumoniae* (6%) (see table 6).

**Table 6:** Number and percentage of organisms grown from aerobic blood culture.

Organism	Total (n = 48)
Non-typhoidal *Salmonella* species [n (%)]	20 (42.6)
*Staphylococcus aureus* [n (%)]	12 (25.5)
*Streptococcus pneumonia* [n (%)]	6 (12.8)
*Escherichia coli* [n (%)]	4 (8.5)
*Proteus species* [n (%)]	1 (2.1)
*Cryptococcus neoformans* [n (%)]	5 (10.6)

Among patients with mycobacterial culture results, 55 (22.1%) of 249 had mycobacteremia. Of these 55 patients, 45.5% had MTB. When compared to patients without MTB positive cultures, patients with MTB positive cultures had higher in-hospital mortality [44.0% (11/25) vs. 22.3% (50/224), *p* = 0.026; OR 1.97, 95% CI = 1.19–3.27] as well as lower median CD4 counts [8.0 vs. 47.0, Mann-Whitney *p* = 0.001].

Approximately 13% (51/379) of patients had malaria parasitemia which mostly (86.3%) consisted of 1+ parasitemia; level of malaria parasitemia was not associated with mortality (data previously shown) [27].

### Laboratory tests

Due to resource constraints, we only assessed metabolic status in the 250 patients enrolled at Mulago. Table 7 shows the relationship of measured laboratory values with respect to in-hospital survival at Mulago Hospital. Multivariate analysis revealed that increased cortisol and decreased albumin levels were independent predictors of in-hospital mortality. None of the laboratory variables were found to predict post-discharge mortality (data not shown).

**Table 7:** Univariate and multivariate analysis for admission laboratory predictors of in-hospital mortality at Mulago Hospital.

	Univariate Predictors			Multivariate Predictors*	
Variable	Death	Survival	*p*	Odds Ratio (95% CI)	*p*
Sodium in mEq/L [mean (SD)]	131.3 (6.7)	132.0 (5.4)	0.47		
Potassium in mEq/L [mean (SD)]	4.2 (0.8)	4.1 (0.6)	0.58		
Chloride in mmol/L [mean (SD)]	95.8 (5.2)	97.3 (4.3)	0.02	—	*n.s.*
Bicarbonate in mmol/L [mean (SD)]	21.1 (3.1)	21.2 (2.9)	0.84		
Blood urea nitrogen in mg/dL [mean (SD)]	10.1 (7.3)	8.2 (8.0)	0.10	—	*n.s.*
Creatinine in umol/L [mean (SD)]	127.4 (105.9)	113.9 (85.6)	0.32		
Glucose in mg/dL [mean (SD)]	122.7 (72.6)	126.2 (66.6)	0.79		
Cortisol in nmol/L [mean (SD)]	425.4 (147.0)	318.5 (133.1)	<0.001	1.01 (1.0–1.01)	<0.001
Albumin in g/dL [mean (SD)]	3.0 (0.5)	3.3 (0.6)	0.002	0.2 (0.1–0.5)	<0.001

* Model is adjusted for covariates found to be significant predictors of in-hospital mortality (i.e., unmarried marital status, decreased admission KPS, and RR>30 breaths/min) in a multivariate analysis for the general Mulago Hospital model (data not shown).

n.s. = not significant.

## Discussion

This study is the first prospective evaluation of the management and outcomes of patients with severe sepsis in a resource-constrained setting. With respect to degree of fluid resuscitation and appropriateness of empiric antibacterials, the management of septic patients in our study was suboptimal and overall mortality was 43%, comprised of both high in-hospital and post-discharge mortality.

While the mortality in this cohort is comparable to mortality reported from cohorts in HICs, it is difficult to compare patients from both settings given stark differences in age distribution. In our study, the predominantly HIV-1 infected participants had a mean age of ∼35 years old with no evidence of cardiovascular comorbidities. In contrast, studies from HICs report patients with a mean age of 65 and multiple cardiovascular comorbidities. Thus, the lower-than-expected mortality in our cohort may suggest that younger patient populations have more robust cardiovascular systems than patients in older cohorts, making them less likely to succumb during their hospitalization when presenting with severe sepsis or septic shock.

Clinical predictors of in-hospital mortality included variables easily measurable in any setting such as morbidity assessment scales (i.e., KPS and GCS) and vital signs (i.e., RR>30 breaths/min). Leukocytosis and thrombocytopenia were also predictive of in-hospital mortality and have been previously shown to be good outcome predictors of patients with septic shock in HIC settings [31]–[33]. Their predictive value in identifying patients at risk for mortality suggests that an admission CBC should be considered a necessary component of initial laboratory investigations for evaluating critically ill patients presenting to resource-constrained hospitals.

Lack of material and human resources contributes to the inability to achieve improved management of sepsis in resource-constrained settings [17]. The high mortality associated with sepsis syndromes in our study may have resulted from this lack of materials and human resources necessary for optimal care. For example, on a 50-bed ward at Mulago Hospital where the number of patients can approach 100, we observed that the nurse to patient ratio was less than 1∶20 and the volume of IV fluids provided to the ward per day was limited to 20 L. In addition, few enrolled patients had access to supplemental oxygen, cardiovascular support medications, or continuous monitoring.

Guidelines used in HICs for the management of severe sepsis recommend that patients receive a minimum initial administration of 20 mL/kg of crystalloid as a fluid challenge with subsequent fluid challenges every 30 minutes for refractory hypotension within the first 6 hours [14]. Although few studies have looked at the direct effect of early fluid resuscitation volumes on clinical outcomes of patients with sepsis, one study reported that septic children receiving 40 mL/kg within the first hour of presentation were significantly less likely to die than those receiving less fluid [34]. Our adult patients (with an estimated mean weight of 50 kg) did not come close to reaching that target, having received less than 2 mL/kg/hr within the first 6 hours of presentation.

While it did not portend improved survival, early fluid resuscitation (i.e., <1hr vs. ≥1 hr) was found to significantly predict mortality in patients discharged from the hospital. Although the reason for this finding is not clear from our data, we found that earlier fluid resuscitation was predicted by measures of illness severity such as admission KPS. These data suggest that sicker-appearing patients were more likely to receive early fluid resuscitation. Even though this early fluid resuscitation may have improved short-term survival for a proportion of patients while hospitalized, these patients were more likely to die soon after leaving the hospital suggesting that the patients' underlying illness was a contributor to their ultimate mortality. Though we did not have complete data on patient comorbidities at the time of admission, our blood culture data from Mulago Hospital suggest a considerable proportion of patients (10%) presenting with sepsis syndromes in Uganda have MTB bacteremia. This information along with the associated high mortality and low CD4 counts highlights a need to improve early case finding of co-infected HIV and MTB patients in highly endemic areas like Uganda.

Antibacterial management also could have been better optimized. Empiric antibacterial therapy was rarely concordant with blood culture sensitivities. The trends we show are consistent with several studies that have shown increased mortality in bacteremic patients receiving inappropriate empiric antibacterials [35], [36].

While a majority of patients received antibacterials, improved survival was neither found in patients receiving early antibacterials nor any regimen of empiric antibacterials. There are several possible reasons for these findings. For one, with the multitude of different antibacterial regimens used, the benefit of an appropriate antibacterial regimen could have been statistically outweighed by the detriment of being administered an inappropriate regimen. In addition, while data suggest that survival of septic patients improves when antibacterials are provided within the first hour of presentation, a small subset (∼1/3) of study patients received their antibacterials within this time frame [37]. Finally, we found variability in the duration and availability of antibacterials when patients are transferred from the Accident and Emergency Department to the medical wards. While we did not collect this information in detail, future studies in these settings are needed to better describe the outcomes of patients with invasive bacterial infections with respect to duration and type of antibacterials.

Due to its affordability in LMICs and its potential for broad spectrum coverage, chloramphenicol is an attractive antibacterial for empiric coverage of bacteremia and has been recommended by the World Health Organization in algorithms for empiric coverage of febrile illnesses [38], [39]. In light of the increased prevalence of non-typhoidal *Salmonella* bacteremia (43% of positive aerobic blood cultures) with the majority of isolates resistant to chloramphenicol, our data along with others suggest that use of chloramphenicol for empiric coverage of Gram negative organisms should be reevaluated [40]. We agree with others who argue for improved microbiologic capabilities in strategically located sentinel hospitals to better characterize local antimicrobial resistance patterns and identify appropriate empiric antibacterial regimens [6], [16].

While our microbiology results are similar to previous studies of bacteremia in hospitalized patients conducted in SSA, a limitation in our study was a low prevalence of culture-positive sepsis (∼20%) [5], [7]–[10], [12], [41], [42]. Notably, low blood culture yields have been shown in sepsis studies in other settings [6], [13], [43]. Additionally, more than half of the study patients (57.7%) reported taking some type of antibacterial prior to hospital admission. Self treatment with easily accessible over-the-counter antimicrobials has been described in this region [44]–[46]. Further research on this type of drug utilization could provide important insight into antibacterial susceptibility patterns in this setting. In addition, although budgetary constraints prevented us from obtaining them, cultures from other sources could have provided more information on microbiologic etiology of patients who presented with a sepsis syndrome despite having negative blood cultures. Given the inability to predict with certainty which patients are likely to succumb to their illness, however, we strongly believe that all patients presenting with the syndrome captured by our inclusion criteria (regardless of culture results) should be ultimately managed early and aggressively in order to avoid death.

Another limitation was the proportion of patients lost to follow up, with 43 of 45 lost patients being from Mulago Hospital. The difference in patients lost to follow-up between sites is likely due to the size of their respective encatchment areas and the ease with which hospital staff can track patients after discharge in a smaller, more rural encatchment area (i.e., MRRH) versus a larger, more urban encatchment area (i.e., Mulago Hospital), especially when patients do not have mobile telephones. Future studies will need to find ways of improving the ability to track patients in order to optimize measurement of survival after patients leave the hospital.

Our study suggests mortality due to severe sepsis was high in a significant proportion of HIV-infected patients admitted to Ugandan medical wards. Furthermore, a considerable number of patients were unaware of their serostatus and only a small percentage was taking highly-active antiretroviral therapy (HAART). These data point to the importance of targeting patients who access hospital-based care for potential HIV treatment and prevention measures. While the benefits of HAART scale-up are becoming increasingly realized in patients who are on therapy, more than 2 million young men and women continue to die annually from HIV-related diseases (e.g., sepsis) [47]. In the current era of HAART scale-up in SSA, improvement in sepsis management may create a window of opportunity for later access to life-saving HIV therapy.

Achieving this goal in settings like Uganda cannot occur unless there is a targeted approach to the management of hospitalized patients with diseases such as sepsis. Thus, focusing on being able to clinically identify this syndrome alongside appropriate management should be paramount during the training and education of all health workers who manage patients similar to those in our study. Such an approach will be most effective alongside the procurement of low cost, high impact and readily available modalities like IV fluids and appropriate antibiotics, improvement in laboratory capabilities, and closer monitoring with increased health personnel.

## References

[1] Vincent JL, Abraham E, Annane D, Bernard G, Rivers E (2002). Reducing mortality in sepsis: new directions.. Crit Care.

[2] Angus DC, Linde-Zwirble WT, Lidicker J, Clermont G, Carcillo J (2001). Epidemiology of severe sepsis in the United States: analysis of incidence, outcome, and associated costs of care.. Crit Care Med.

[3] Martin GS, Mannino DM, Eaton S, Moss M (2003). The epidemiology of sepsis in the United States from 1979 through 2000.. N Engl J Med.

[4] Hotchkiss RS, Karl IE (2003). The pathophysiology and treatment of sepsis.. N Engl J Med.

[5] Archibald LK, den Dulk MO, Pallangyo KJ, Reller LB (1998). Fatal Mycobacterium tuberculosis bloodstream infections in febrile hospitalized adults in Dar es Salaam, Tanzania.. Clin Infect Dis.

[6] Archibald LK, Reller LB (2001). Clinical microbiology in developing countries.. Emerg Infect Dis.

[7] Archibald LK, McDonald LC, Nwanyanwu O, Kazembe P, Dobbie H (2000). A hospital-based prevalence survey of bloodstream infections in febrile patients in Malawi: implications for diagnosis and therapy.. J Infect Dis.

[8] Arthur G, Nduba VN, Kariuki SM, Kimari J, Bhatt SM (2001). Trends in bloodstream infections among human immunodeficiency virus-infected adults admitted to a hospital in Nairobi, Kenya, during the last decade.. Clin Infect Dis.

[9] Peters RP, Zijlstra EE, Schijffelen MJ, Walsh AL, Joaki G (2004). A prospective study of bloodstream infections as cause of fever in Malawi: clinical predictors and implications for management.. Trop Med Int Health.

[10] Gilks CF, Brindle RJ, Otieno LS, Simani PM, Newnham RS (1990). Life-threatening bacteraemia in HIV-1 seropositive adults admitted to hospital in Nairobi, Kenya.. Lancet.

[11] Grant AD, Djomand G, De Cock KM (1997). Natural history and spectrum of disease in adults with HIV/AIDS in Africa.. Aids.

[12] Gordon MA, Walsh AL, Chaponda M, Soko D, Mbvwinji M (2001). Bacteraemia and mortality among adult medical admissions in Malawi–predominance of non-typhi salmonellae and Streptococcus pneumoniae.. J Infect.

[13] Rivers E, Nguyen B, Havstad S, Ressler J, Muzzin A (2001). Early goal-directed therapy in the treatment of severe sepsis and septic shock.. N Engl J Med.

[14] Dellinger RP, Levy MM, Carlet JM, Bion J, Parker MM (2008). Surviving Sepsis Campaign: international guidelines for management of severe sepsis and septic shock: 2008.. Critical Care Medicine.

[15] Becker JU, Theodosis C, Jacob ST, Wira CR, Groce NE (2009). Surviving sepsis in low-income and middle-income countries: new directions for care and research.. Lancet Infect Dis.

[16] Petti CA, Polage CR, Quinn TC, Ronald AR, Sande MA (2006). Laboratory medicine in Africa: a barrier to effective health care.. Clin Infect Dis.

[17] Dunser MW, Baelani I, Ganbold L (2006). A review and analysis of intensive care medicine in the least developed countries.. Crit Care Med.

[18] Theodosis C, Brenner S (2006). Framework and Rationale for Studying Sepsis in High HIV Seroprevalence Resource Poor Settings: the Livingstone General Hospital Experience..

[19] Blomberg B, Manji KP, Urassa WK, Tamim BS, Mwakagile DS (2007). Antimicrobial resistance predicts death in Tanzanian children with bloodstream infections: a prospective cohort study.. BMC Infect Dis.

[20] Cheng AC, West TE, Limmathurotsakul D, Peacock SJ (2008). Strategies to reduce mortality from bacterial sepsis in adults in developing countries.. PLoS Medicine.

[21] Rivers EP, McIntyre L, Morro DC, Rivers KK (2005). Early and innovative interventions for severe sepsis and septic shock: taking advantage of a window of opportunity.. CMAJ.

[22] Wanyenze R, Kamya M, Liechty CA, Ronald A, Guzman DJ (2006). HIV counseling and testing practices at an urban hospital in Kampala, Uganda.. AIDS Behav.

[23] Bone RC, Balk RA, Cerra FB, Dellinger RP, Fein AM (1992). Definitions for sepsis and organ failure and guidelines for the use of innovative therapies in sepsis. The ACCP/SCCM Consensus Conference Committee. American College of Chest Physicians/Society of Critical Care Medicine.. Chest.

[24] Mor V, Laliberte L, Morris JN, Wiemann M (1984). The Karnofsky Performance Status Scale. An examination of its reliability and validity in a research setting.. Cancer.

[25] Karnofsky DA (1961). Meaningful clinical classification of therapeutic responses to anticancer drugs.. Clin Pharmacol Ther.

[26] Erikstrup C, Kallestrup P, Zinyama R, Gomo E, Mudenge B (2007). Predictors of mortality in a cohort of HIV-1-infected adults in rural Africa.. J Acquir Immune Defic Syndr.

[27] Moore CC, Jacob ST, Pinkerton R, Banura P, Meya DB (2009). Treatment of severe sepsis with artemether-lumefantrine is associated with decreased mortality in Ugandan patients without malaria.. Am J Trop Med Hyg.

[28] Moore CC, Jacob ST, Pinkerton R, Meya DB, Mayanja-Kizza H (2008). Point-of-care lactate testing predicts mortality of severe sepsis in a predominantly HIV type 1-infected patient population in Uganda.. Clinical Infectious Diseases.

[29] Health Management Information (HMIS) Database Mulago Hospital Complex (2008)

[30] Health Management Information (HMIS) Database Masaka Regional Referral Hospital Complex (2008)

[31] Alberti C, Brun-Buisson C, Goodman SV, Guidici D, Granton J (2003). Influence of systemic inflammatory response syndrome and sepsis on outcome of critically ill infected patients.. Am J Respir Crit Care Med.

[32] Sharma B, Sharma M, Majumder M, Steier W, Sangal A (2007). Thrombocytopenia in septic shock patients–a prospective observational study of incidence, risk factors and correlation with clinical outcome.. Anaesth Intensive Care.

[33] Martin CM, Priestap F, Fisher H, Fowler RA, Heyland DK (2009). A prospective, observational registry of patients with severe sepsis: the Canadian Sepsis Treatment and Response Registry.. Crit Care Med.

[34] Carcillo JA, Davis AL, Zaritsky A (1991). Role of early fluid resuscitation in pediatric septic shock.. JAMA.

[35] Bochud PY, Glauser MP, Calandra T (2001). Antibiotics in sepsis.. Intensive Care Med.

[36] Leibovici L, Shraga I, Drucker M, Konigsberger H, Samra Z (1998). The benefit of appropriate empirical antibiotic treatment in patients with bloodstream infection.. J Intern Med.

[37] Kumar A, Roberts D, Wood KE, Light B, Parrillo JE (2006). Duration of hypotension before initiation of effective antimicrobial therapy is the critical determinant of survival in human septic shock.. Crit Care Med.

[38] WHO (2007). Clinical Management of HIV and AIDS at District Level.. http://www.searo.who.int/LinkFiles/Publications_ch9.pdf.

[39] (2004) Integrated Management of Adult and Adolescent Illness: Interim Guidelines for First-Level Facility Health Workers at Health Centre and District Outpatient Clinic..

[40] Gordon MA, Graham SM, Walsh AL, Wilson L, Phiri A (2008). Epidemics of invasive Salmonella enterica serovar enteritidis and S. enterica Serovar typhimurium infection associated with multidrug resistance among adults and children in Malawi.. Clin Infect Dis.

[41] Ssali FN, Kamya MR, Wabwire-Mangen F, Kasasa S, Joloba M (1998). A prospective study of community-acquired bloodstream infections among febrile adults admitted to Mulago Hospital in Kampala, Uganda.. J Acquir Immune Defic Syndr Hum Retrovirol.

[42] McDonald LC, Archibald LK, Rheanpumikankit S, Tansuphaswadikul S, Eampokalap B (1999). Unrecognised Mycobacterium tuberculosis bacteraemia among hospital inpatients in less developed countries.. Lancet.

[43] Finfer S, Chittock DR, Su SY, Blair D, Foster D (2009). Intensive versus conventional glucose control in critically ill patients.. N Engl J Med.

[44] Ogwal-Okeng JW, Obua C, Waako P, Aupont O, Ross-Degnan D (2004). A comparison of prescribing practices between public and private sector physicians in Uganda.. East Afr Med J.

[45] Awad AI, Eltayeb IB (2007). Self-medication practices with antibiotics and antimalarials among Sudanese undergraduate university students.. Ann Pharmacother.

[46] Anyama N, Adome RO (2003). Community pharmaceutical care: an 8-month critical review of two pharmacies in Kampala.. Afr Health Sci.

[47] 2008 Report on the global AIDS epidemic (2008). Status of the global AIDS epidemic. Geneva: Joint United Nations Programme on HIV/AIDS (UNAIDS), 2008..

